# Quantitatively evaluating the cross-sectoral and One Health impact of interventions: A scoping review and case study of antimicrobial resistance

**DOI:** 10.1016/j.onehlt.2020.100194

**Published:** 2020-11-14

**Authors:** Nichola R. Naylor, Jo Lines, Jeff Waage, Barbara Wieland, Gwenan M. Knight

**Affiliations:** a London School of Hygiene & Tropical Medicine, London, United Kingdom; b International Livestock Research Institute (ILRI), Addis Ababa, Ethiopia

**Keywords:** One Health, Antimicrobial resistance, Economic evaluation, Impact evaluation, AMR, Antimicrobial resistance, DALY, Disability Adjusted Life Year, GDP, Gross Domestic Product, NEOH, Network for Evaluation of One Health, MCDA, Multi-criteria decision analysis, OH, One Health

## Abstract

**Background:**

Current frameworks evaluating One Health (OH) interventions focus on intervention-design and -implementation. Cross-sectoral impact evaluations are needed to more effectively tackle OH-issues, such as antimicrobial resistance (AMR). We aimed to describe quantitative evaluation methods for interventions related to OH and cross-sectoral issues, to propose an explicit approach for evaluating such interventions, and to apply this approach to AMR.

**Methods:**

A scoping review was performed using WebofScience, EconLit, PubMed and gray literature. Quantitative evaluations of interventions that had an impact across two or more of the human, animal and environment sectors were included. Information on the interventions, methods and outcome measures found was narratively summarised. The information from this review informed the construction of a new approach to OH-related intervention evaluation, which then was applied to the field of AMR.

**Results:**

The review included 90 studies: 73 individual evaluations (from 72 papers) and 18 reviews, with a range of statistical modelling (*n* = 13 studies), mathematical modelling (*n* = 53) and index-creation/preference-ranking (*n* = 14) methods discussed. The literature highlighted the need to (I) establish stakeholder objectives, (II) establish quantifiable outcomes that feed into those objectives, (III) establish agents and compartments that affect these outcomes and (IV) select appropriate methods (described in this review) accordingly. Based on this, an evaluation model for AMR was conceptualised; a decision-tree of intervention options, a compartmental-microeconomic model across sectors and a general-equilibrium (macroeconomic) model are linked. The outcomes of this multi-level model (including cost-utility and Gross Domestic Product impact) can then feed into multi-criteria-decision analyses that weigh respective impact estimates alongside other chosen outcome estimates (for example equity or uncertainty).

**Conclusion:**

In conclusion, stakeholder objectives are key in establishing which evaluation methods (and associated outcome measures) should be used for OH-related interventions. The stated multi-level approach also allows for sub-systems to be modelled in succession, where resources are constrained.

## Highlights

• Quantitative impact evaluations of One Health interventions are vital.
• We provide a compendium of evaluations of cross-sectoral interventions.
• Decision makers' objective functions are key in selecting methods.
• For antimicrobial resistance, a multi-level, compartmental model is suggested.

## 1 Introduction

Antimicrobial resistance (AMR) may reduce our ability to prevent and treat infections in humans and animals [1]. It has been described as a true One Health (OH) issue [[2], [3], [4]], in which OH can be defined as the description of, and interactions between, the individual, population and ecosystem levels of health (across humans, animals, plants and the wider environment) [5]. For the purposes of this paper, ‘cross-sectoral’ relates to the interaction between two or more of these ecosystem factors (human, animal and ‘plants and environment’).

Policy options to tackle the emergence and spread of AMR have been put forward through international policy reports and action plans [6,7].While there is some evidence of a positive effect of reducing antimicrobial use in food-producing animals on AMR outcomes in humans [8], there is a lack of evidence that quantifies the wider socio-economic and OH impact [9,10]. The Network for Evaluation of One Health (NEOH) is one of various initiatives offering frameworks for evaluation in OH topics [11,12]. However, the NEOH do not propose a specific framework for how to perform economic impact evaluations of AMR interventions from a OH-perspective. Methods for evaluating AMR-burden and related intervention impact have been proposed [10], but mainly from a human-health perspective.

In the OH literature, two key literature reviews highlight potential quantitative outcomes useful in evaluating OH interventions. However, these reviews do not discuss in detail methods of how to model cost outcomes nor offer an explicit approach to cover multiple outcomes [13,14]. Given the complexity of integrating OH evaluations across different sectors, existing economic evaluation checklists do not offer an appropriate discussion of the health and/or economic impact of cross-sectoral interventions [15]. Such discussion on complex, OH intervention evaluation is needed for the field to obtain robust estimates of resulting intervention impacts.

It has previously been highlighted that AMR poses similar theoretical evaluation issues to climate change and zoonotic infections, due to their cross-sectoral nature and/or link with greater societal costs due to individual behaviours [[16], [17], [18]]. Hence there is scope to learn from, and adapt, existing cross-sectoral evaluation approaches from these topics within the field of AMR.

This study therefore aims to (i) collate and describe previous methods used in the quantitative evaluation of interventions related to OH and other cross-sectoral issues, (ii) offer an explicit approach for evaluating such interventions, and (iii) apply this approach to the case of AMR-related interventions.

## 2 Materials and methods

A scoping review allows for the identification of main concepts within a particular area of interest [19]. Therefore, a scoping review method was used to achieve our first objective. Retrievals that the authors thought were likely to provide a high inclusion rate of relevant literature (such as OH surveillance and climate change [18])) were sought. Within WebofScience, EconLit and Google the following search strings were used [[20], [21], [22]]; (1) (“One Health” AND “evaluation”), (2) (“economic” AND “evaluation” AND “health” AND “agriculture”), (3) (“economic” AND “evaluation” AND “climate change”). An additional search was conducted in PubMed utilising (“One Health” AND surveillance AND economic evaluation) [23], with 28th March 2019 being the last search date for all searches. A protocol for this study was not published, but the reporting of this scoping review is aligned with the PRISMA-ScR checklist (see Appendix A).

Titles were reviewed by the lead author, followed by abstracts (if available), and subsequently full texts (if available). A study was included if it contained quantitative impact estimates across more than one sector (human, animal and environment) within the intervention evaluation. See Table 1 for the inclusion and exclusion criteria applied. Reference lists were also used to identify additional literature.

**Table 1:** Inclusion/exclusion criteria applied in the scoping review. Layout using a PICOS criteria approach [24], with the addition of the “Study Type” and “Language” categories.

Criteria	Inclusion	Exclusion
Population	Human, animal, agriculture, environment, economy	No outcomes or evaluation including at least two of the stated populations of interest (human, animal, agriculture, environment, economy)
Intervention	An intervention that is aimed at tackling a One Health or cross-sectoral issue	No specific intervention or policy
Comparator	Standard practice/Do nothing/business-as-usual	No exclusion criteria applied
	Alternative interventions/policy scenarios	
Outcome	Quantitative outcome	No quantitative outcomes
		Quantitative outcomes in only one sector (e.g. only energy costs estimated in a policy aimed at energy)
Study Design	Economic Model	No specific exclusion criteria applied
	Mathematical Model	
	Statistical Model	
	Observational study (randomised controlled trial, case-control or cohort)	
	Review (separately included from individual studies)	
Study type	Peer-reviewed Publication	Letters
	Reports	Case studies (descriptive)
	Book and/or Book Chapter	Conference Abstracts
		Protocol
Language	English	

For each evaluation study, the OH issue, intervention, spatial and temporal scope, main method, main data source, key inputs, key outputs (monetary and non-monetary) and potential limitations were noted using a data extraction table (see Appendix D). Summary indicators were used (see Table 2). Relevant literature reviews were included, with their aims and results summarised separately (see Appendix C). Due to the research aims, and study design heterogeneity, no risk of bias checklist was used.

**Table 2:** Scoping review indicator definitions.

Indicator⁎	Classification	Definition
One Health perspective	Human	Impact quantified on a person; including patients, consumers and farmers within the system under evaluation.
	Animal	Impact quantified on animals; including livestock, fish, companion animals.
	Environment	Impact quantified on the environment, including on temperature, water levels and on crops.
Evaluation Perspective	Individuals	Evaluating impact on health burden and income at the individual level.
	Microeconomic (Firm and Sector)	Evaluating impact within one specific sector; such as health care, environmental and agricultural sectors individually. It also included individual business impact, such as farm-level impact.
	Macroeconomic (Multi-sector and Government)	Evaluating impact across multiple sectors within an economy or globally.
Methodology perspective	Mathematical Simulation	Methods which take a hypothetical sample and model potential interactions and/or outcomes using mathematical formulae. This ranges from simple stepwise calculation methods (e.g. applying prevalence levels to a population of interest) to complex system dynamic models and general computable equilibrium models [25].
	Statistical Evaluation	Methods which take empirical data and apply statistical methods to estimate interactions and/or associated outcomes. This ranges from the calculation of descriptive statistics to complex survival analyses and regression analyses [25].
	Index/Rank Creation & Calculation	Methods which utilise a framework to compile an index to measure the intervention, or a formalised ranking system. For example, multi-criteria decision analyses [26].
	Other	If the study method did not fit into any of the above methodology perspective categories, then Other was used.

⁎ These refer to the intervention evaluation perspective.

Results are presented descriptively, with key concepts found highlighted. This is then used to propose an explicit approach for evaluating OH interventions. This proposed approach was then applied to AMR-interventions, along with AMR-based literature, to construct a conceptual evaluation model. A visual schematic of this conceptual model was drafted and narratively discussed.

## 3 Results

### 3.1 Literature characteristics

1479 unique retrievals followed by 168 full texts were screened from the formal searches, leading to 65 studies being included. The majority of texts were excluded due to either not evaluating an intervention/policy or only evaluating within one sector. Through reference lists and Google searches, an additional 25 papers were added. 72 evaluation studies (73 individual evaluations) and 18 literature reviews were included. All individual studies included human impact (73 evaluations), with more including environmental than animal impact (see Appendix B). Only 29 (40%) of the 73 evaluations included all 3 perspectives. The majority of studies covered only one evaluation perspectives (39/73), usually either micro- or macroeconomic, with 29 having two evaluation perspectives and 5 studies covering all three. Interventions were tackling issues associated with climate change (38/73), zoonotic infections (21/73), antimicrobial resistance (1/73) or other cross-sectoral issues (11/73).

### 3.2 Frameworks & approaches proposed and used in cross-sectoral evaluation

A number of the reviews offered a guide/framework to the approach of cross-sectoral evaluation [12,16,[27], [28], [29]]. Previous reviews highlight the need to involve stakeholders throughout the evaluation process, and tailor evaluations according to stake-holder objectives which have been defined clearly (e.g. maximizing population health or monetary gains) [16,25,28,[30], [31], [32], [33]]. One review on the use of ‘farm models’ in policy (for example looking at the economic, environmental and/or social impact of different Common Agricultural Policy initiatives) highlighted the importance of considering the end-user and involving stakeholders when developing such models, however it found that only 23% of the 184 studies reported stakeholder consultation [30].

Other concepts highlighted through previous reviews and proposed frameworks include; incorporating all plausible scenarios [12,16,25,29], stress-testing models with extreme scenarios [16,27,29], allowing for iterations over-time [29,32,34], quantifying distributions of effects [13,35,36] and explicitly discussing uncertainty [29,33,37].

### 3.3 Outcome measures used in cross-sectoral evaluation

Out of the 73 individual studies that underwent data extraction, 52 (72%) and 45 (62%) had main outcomes of non-monetary and monetary valuations respectively. 24 of these (33%) included both, with 6 including impact on human health burden and monetary outcomes [17,[38], [39], [40], [41], [42]]. Many of these were climate change/emissions related interventions (15/24) that included an emissions (environmental) impact and a subsequent cost/Gross Domestic Product (GDP) Impact.

Morbidity measures were based on utility and welfare (such as animal welfare scores for animal impact [40]). Disability Adjusted Life Years (DALYs) were used in some evaluations, combining both mortality and morbidity impacts in relation to human impact [17,40,42,43]. Cost per DALY averted outcomes were compared to stated monetary, willingness-to-pay thresholds (such as $30,000 per DALY averted) to conclude whether animal vaccination was cost-effective from a OH perspective in two of the studies found [17,43]. In some of the environmental science literature, monetary values of health outcomes were termed as the “value for statistical life” (such as 40,000 EUR per loss of life year [44]) [39]. Discussion on how to elicit or calculate ‘willingness-to-pay thresholds’ and monetary life-year values is available [33,45]. Cost-benefit and cost-utility outcomes were also highlighted in included literature reviews (see Appendix C) [13,14,27,30,36].

Other potentially important monetary outcomes emphasised by previous reviews include profit, income, stakeholder cost-savings and productivity valuations [13,14,27,30,33]. Whilst human, social and economic development indicators, environmental sustainability, biodiversity, equality, equity and ethical implications were highlighted as key non-monetary outcomes [13,14,27,30,33,34,36].

### 3.4 Methods used in cross-sectoral evaluation

Thirteen studies used statistical evaluation techniques; including regression modelling [46,47], trend analysis [48,49] and basic descriptive statistics [50,51] to estimate the size of the intervention impact or summarise outcome measures on a particular population, respectively. Estimates of different levels of burden (from different perspectives) based on statistical analyses can feed into mathematical model-based evaluations [25], such as that used for the economic evaluation of climate protection measures in Germany [52]. This study performed econometric evaluations of energy (e.g. price) and economy (e.g. national account) data to estimate mathematical model parameter values that fed into structural equations. This allowed them to forecast the impact of interventions, such as the ‘Energy Transformation Scenario’, on GDP and related components (e.g. sector employment).

The majority of studies analysed (53/73) used mathematical simulations to estimate cross-sectoral impact, which included not only cost-benefit and cost-utility analyses (10/73), but also basic calculations, computable general equilibrium and systems dynamics models (see Appendix D).

Hutton suggests performing both cost-benefit and cost-utility analyses simultaneously, providing both cost-benefit ratios and cost per DALY averted respectively for relevant stakeholders [33]. One microeconomic evaluation study which provided both monetary and non-monetary outcomes for a range of stakeholder perspectives was on rabies vaccination [40]. This study separated potential outcome measures useful to different decision-makers, such as monetary expenditure, DALYs averted, animal welfare and dog acceptance (the latter two being “qualitative scores”) and estimated uncertainty through probabilistic sensitivity analysis [40]. In contrast, few of the studies found using computable general equilibrium analyses and systems dynamics models explicitly quantified uncertainty, instead including scenario analyses only [53,54]. The ECONADAPT toolbox provides a useful list of methods that explicitly incorporate uncertainty [37].

Finally, fourteen studies used ‘index creation’ methods (see Table 2 for definition), with six using multi-criteria decision analysis (MCDA) and 8 using “One Health-ness” indices [[55], [56], [57], [58], [59], [60], [61], [62]]. The latter may be useful if a stakeholder views ‘OH-ness’ as an important factor within their assessment of the effectiveness of interventions [12]. One example of MCDA application was in Lyme disease management strategies [63], where multiple interventions were ranked based on public health, animal, environmental and social impact as well as economic, strategic, operational and surveillance criteria [63]. However, in order to rank policies based on criteria such as “reduction on incidence of human cases” and “impact on cost to public sector”, the outputs of epidemiological and health economic models are needed. In this example, these outcomes were estimated through literature reviews and/or expert panels [63]. MCDA methods can also explicitly incorporate equity and/or uncertainty impacts of interventions [37,64].

Aside from the aforementioned methods of modelling, the scoping review also found scope for linking and extending previous mathematical and economic models within the field of climate change mitigation intervention evaluation [52]. An example is the dynamic climate-economic computable general equilibrium model (‘GDynE’). This was previously adapted from a similar previous model (‘GDyn’), was linked with the ‘Global Trade Analysis Project -Power’ database to create a computable general equilibrium model and a cost-benefit analysis to estimate the global net monetary impact of climate change mitigation policy [65]. Another study applied the NAADSM (North American Animal Disease Spread Model) to Influenza transmission across swine and humans in Canada [66].

### 3.5 Assumptions in cross-sectoral evaluation

Most studies included in this review had a time-horizon (the length of time of the evaluation) of less than 50 years (15/73 studies used less than or equal to one year, 35/73 between 10 and 50 years). Those that had longer time-horizons (14/73 studies had 50+ years^1^) tended to have a macroeconomic approach and use computable general equilibrium, cost-benefit or MCDA approaches. The majority of papers that had a time horizon of less than 10 years were using statistical evaluations of data or collecting information to create indices. Such studies are generally limited by the time period of data collection. Further use of time-series data across a longer time-horizon would be useful in understanding and forecasting the longer-term burden associated with interventions targeting such issues [67].

A discount rate is a factor which accounts for stakeholders' time preference. This factor allows users to value future benefits/costs (for example healthcare costs in the year 2050) in today's` values [68]. Reviewed studies used discount rates ranging from 1% to 9% [69,70], with some utilising a social discount rate which varied over time [27,71] and some testing multiple discount rates through scenario analyses [70,72,73].

### 3.6 An approach for One Health intervention impact evaluation

The review highlighted that to evaluate OH intervention impact one should (1) define the evaluation-related research question explicitly (including all relevant scenarios), (2) define the system (inputs, outputs and interactions) and theory of change, and (3) consider appropriate methods that capture the whole system that is impacted by changes through the intervention, and value outcomes accordingly [12,16,27,28]. We build on this by specifying a framework to perform part (3), broken down into steps (I) to (IV) below.

(I) Establish stakeholder objectives (‘objective functions’): In Table 3 we list possible objectives of these different agents within a potential OH system, drawing on examples from the wider literature. The main economic theories used in the selection of potential objectives in Table 3 are grounded in utilitarianism (maximizing overall ‘satisfaction’) as the main social justice perspective. However, the possibility to include egalitarian motives or capability approaches (focusing on equality and capability across populations respectively) is also noted [74]. Uncertainty was highlighted in previous reviews as something that should be explicitly considered and quantified, as this has been consistently stated as being valued by decision makers [30,32,33,64]. Ideally this would be performed with the involvement of stakeholders (e.g. through formal stakeholder participation methods that can deal with conflicting objectives [75]).

(II) Establish what components of those objective functions should be estimated: Based on the research question, system, theory of change and objective function definitions, the components of the objective functions that may be impacted by the intervention can be highlighted. By providing outcome estimates (such as net income impact) that feed into stakeholder objective functions (such as net income maximisation), the intervention evaluation provides information that can then aid changes in decision making (such as whether to uptake, continue or stop use of an intervention as it reduces net income).
(III) Establish which agents from the system (such as patients/farmers) and/or compartments (such as human/animal health states) may affect these outcomes.
(IV) Select methods that allow for the modelling of interactions between agents, compartments and selected outcomes. Things to consider here include:
  a. If it is a large system with many agents and/or compartments, can it be broken down into sub-systems? These can then be linked to other available models or expanded on in the future.
  b. Within the chosen system, which modelling method is robust and feasible? Potential modelling methods and limitations are discussed in the scoping review (see study methods described in Appendices C and D).
  c. Are data sources available and accessible for associated statistical analyses and/or literature available for parameterisation of such a model?

### 3.7 A case study: antimicrobial resistance and associated interventions

We apply the above framework to AMR interventions in order to provide a clear example of use of the framework. We do not specify the intervention itself but take a broader AMR overview.

(1) Define Research aim:

To estimate the impact of an intervention aimed at tackling AMR. In practice, this would be defined by many specifics, with a linked antimicrobial, species, infection type etc.

**Fig. 1:**
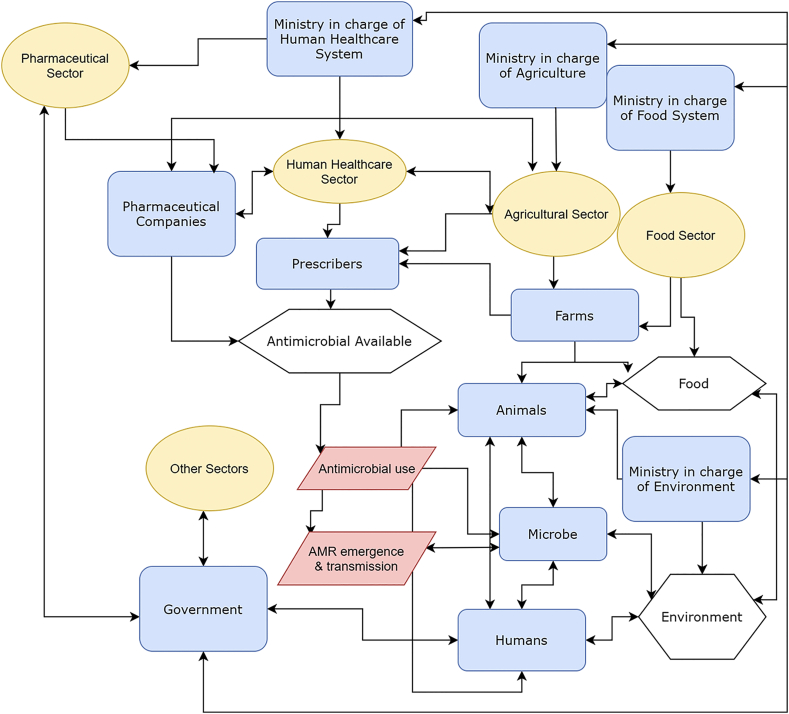
The system under evaluation for cross-sectoral an antimicrobial resistance intervention: Adapted from Ruegg et al [79] (Fig. 2). Ovals represent sectors, boxes represent agents, hexagons represent resources and parallelograms represent actions related to antimicrobial stewardship. Connecting lines represent potential relationships related to the issue and intervention. ‘Ministry’ may be multiple ministries in reality (for example, food system may include commerce and additional governmental offices). AMR: antimicrobial resistance.

(2) Define System and theory of change:

A published systems map of ‘resource systems’, ‘resource units’ and ‘governing systems’ for AMR was adapted [79]. Our adaptation refocuses the system to define agents, actions, resources and sectors [79] in line with our proposed framework (see Fig. 1). For example, one intervention could be antimicrobial stewardship with a simplified theory of change that stewardship reduces antimicrobial usage, which in turn reduces AMR emergence.

(3) Select methods for intervention impact evaluation:

(I) & (II) Establish stakeholder objective functions and outcomes:

Through combining Fig. 1 (outlining stakeholders) and Table 3 (outlining potential objective functions), we provide potential factors and outcomes in Table 4. Uncertainty across all the individual components is suggested to be quantified where possible.

(III) Establish from the system which agents and/or compartments affect selected outcomes:

**Table 3:** Potential economic agent objectives and constraints.

Agent	Potential objectives and constraints	Example(s) from the literature
Individuals	Individuals may seek to maximize individual expected utility over their lifetime (or over other pre-defined time horizons), based on savings, consumption of commodities and consumption of leisure. This includes the consumption of healthcare. This will be subject to budget constraints (a function of income). Broadening this, individuals could also seek to maximize individual capability [74].	*Smith* et al. directly define a utility maximisation problem using equations involving utility values attributed to the consumption of commodities and leisure, solved using computable general equilibrium techniques [76].
Firm	Firms may seek to maximize utility (a function of profits), based on the consumption of labour, capital and intermediate inputs, subject to the price of the inputs and output. However, risk or uncertainty may also factor into decision making processes of firms.Time horizons, over which a firm's objective function is maximised, will depend on the nature of the production process and individual firms (for example this could be per quarter, financial year or crop cycle).	A review of farm-level evaluations found that half of the studies used profit maximisation as the objective function, 29% included risk or stochasticity, whilst 18% of the studies used multi-criteria objective functions (including income, risk and labour factors) [30].A study on water resource management adaptation policies discussed how utility maximisation is often equated to profit maximisation for firms. However this study proposes using a multi-attribute utility maximisation when using a firm perspective, with factors such as risk avoidance and management complexity included [77].
Sector	These decision makers may be attempting to maximize the return on an investment within that sector. This could involve maximizing the expected health-related quality of life or monetary return of a given investment, or maximizing productivity (rate of such outputs for a given set of inputs), constrained by different financing issues depending on the sector and its context (e.g. public versus private). These factors will also impact the desired time horizon for which the objective maximisation process is considered.Increasing population capability could alternatively be the motivation, either through maximisation of total capability or through meeting a threshold level of capacity for as many people in society as possible [74].	By using cost-utility analyses, such as estimating cost per disability-adjusted life year averted [43], studies assumed that payers want to maximize expected population utility, subject to budget constraints.In cost-benefit analyses which apply a set monetary value to life/utility, there is an assumption that a positive return on investment is the goal of the intended sector [44].
Government	A general objective function proposed for a nation's government is that of maximizing government utility from the consumption of commodities, capital and labour, constrained by tax revenues [76], and loans or other financing mechanisms. Government utility, however, could encompass wider social preferences in the form of being a function of equity, capability and risk/uncertainty.Chosen time horizons and time preference assumptions may differ depending on policy perspectives and external forces (such as political election or budgetary cycles).	Previous reviews highlighted the importance of outcomes such as equity, capability, sustainability, uncertainty and animal welfare [13,30,33,64]. A social cost-benefit analysis included various social discount rates alongside a survey on what the appropriate discount rate would be [71].Economic agent objectives were incorporated into a climate change policy evaluation found in this review in the form of a ‘multi-level model’. This allowed for an international level and lower levels to be linked and was based on a game of negotiations, using an underlying multi-attribute utility function [78]. One review found highlighted the lack of agricultural/food-security evaluations that included potentially important criteria such as economic development and environmental sustainability [36].

**Table 4:** Potential Objective Function Factors and Related Outcomes for the Case Study.

Stakeholder	Objective function factor	Measurable outcome
Individuals (patient, the public)	• Net income • Utility	• Employment rates • Per capita net income • Mortality • Utility (e.g. Quality-adjusted life year)
Firms (farm, pharmaceutical company)	• Income • Revenue • Profit • Risk	• Firm income, costs, profit • Firm productivity • Cost-benefit
Sector – Human Healthcare System (e.g. Minister)	• Cost • Mortality • Morbidity/Utility • Budget	• Mortality rates and/or case fatality rates • Infection epidemiology • Cost-effectiveness • Cost-utility • Budget-impact
Sectors – Agriculture and Food Systems	• Cost • Sector productivity • Budget • Nutrition	• Cost-benefit • Productivity • Mortality rates and/or case fatality rates • Infection epidemiology • Cost-utility related to malnutrition
Sector – Environmental System	• Resource availability • Pollution • Biodiversity	• Environmental contamination (e.g. through residues or resistant microbes)
Government	• National productivity and accounts • Population utility • Cost-benefit • Equity • Risk	• Gross domestic Product • Population mortality & morbidity • Infection epidemiology • Environmental resource

**Fig. 2:**
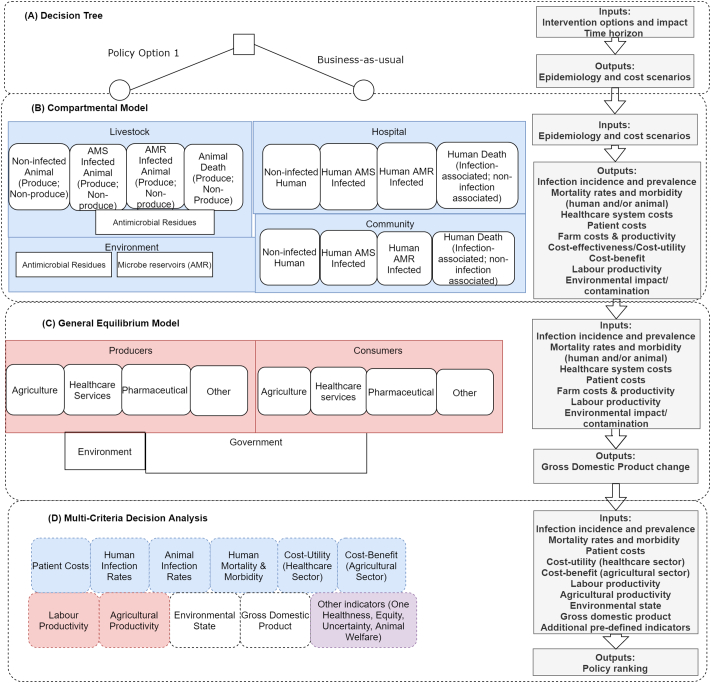
A conceptual multi-level model for evaluating cross-sectoral antimicrobial resistance interventions. White boxes represent health states or sector states. Segments (A) to (D) represent the model method. Shaded boxes represent settings in (A) – (C) and respective model results in (D). Transitions can occur between white boxes within each segment (including across setting), such as from animal antimicrobial susceptible carrier to antimicrobial susceptible human carrier within (B), but these lines have not been added for visual simplicity. Inputs refer to those changed through the intervention and not all model inputs. Abbreviations: AMR – antimicrobial resistance, AMS – antimicrobial susceptible,

Through Fig. 1, Tables 1 and 4, we can see that the general public, famers, prescribers, ministries across OH and the government in general may affect outcomes. The health state of humans and animals, which is affected by antimicrobial availability, use and AMR, in turn effects sector costs and productivity. Pharmaceutical company profit calculations are business-sensitive information and evaluation methods that feed into their decision-making processes were beyond the scope of this paper.

(IV) Selecting an appropriate modelling methodology:

Previous methods used within and across sectors in evaluating AMR and AMR-interventions have been discussed [80], with relevant papers cited throughout this section. From the initial conceptual evaluation illustrated in Fig. 2, we can see which parameters are needed for the proposed decision tree (A) and compartmental model (B). Statistical data analyses could be used to parametrise important factors such as: trends in resistance levels, demography of the populations of interest, specific factor productivity levels and outcomes associated with antimicrobial susceptible and resistant infections (across humans and animals) [[81], [82], [83]]. Data on antimicrobial usage, resistance and patient outcomes are becoming more available [82,84,85].

The selection of models depicted in Fig. 2(B) and (C) allows for the outputs of one model to become the inputs of the next. The compartmental model structure in Fig. 2(B) also allows for the emergence and transmission of AMR to be modelled with the attachment of monetary benefits to the human healthcare system (such as applying a monetary value per life years lost) [86,87], allowing for an aggregation of monetary costs and benefits across human healthcare and livestock systems, giving a cross-sectoral cost-benefit estimate. Additionally, DALYs (or Quality Adjusted Life Years) can be attached to give cost-utility values [87]. Humans or animals may transition across any of the health states within each segment (Fig. 2(B)). However, in the general equilibrium model, the compartments are agent states for which changes of economic inputs (e.g. labour) and outputs (e.g. product) occur through mathematical functions for definitions of agent behaviour [88,89], scenario analyses (with stress-testing) and sensitivity analyses can be used to quantify methodological and parameter uncertainty [90]. Such methods can be extended to also look at poverty impacts [88].

Given that the implementation of AMR policy is over multiple years and affects multiple cohorts, the evaluation model could be multi-cohort (i.e. not just following one “average” cohort of humans and animals over time). Sub-group analyses across the model would allow for equity measures to be available for the MCDA stage of evaluation. The life-expectancy of the population included within the models is suggested to be the model time-horizon, however outcomes can be estimated at earlier time points if needed according to the decision-maker's objective functions. Appropriate discount rates and willingness-to-pay thresholds should be used accordingly [91].

The proposed structure allows for different levels to be built concurrently or subsequently by multiple parties. Allowing for a structured integration of knowledge accumulation and parameterisation over time. Utilising the model-sharing and -adaptation approach seen in the climate change literature, this could be feasible to complete in the medium term. Once built, the model could also be expanded to allow more complex structures and feedback mechanisms to be integrated, such as those in system dynamics models [92].

## 4 Discussion

We utilise evidence from 90 previous studies and reviews to describe previous methods used in evaluating interventions across sectors. Of the 73 individual evaluations included, the majority of studies; (i) focused on only two sectors, (ii) only had one evaluation perspective (e.g. microeconomic) and (iii) only had either monetary or non-monetary outcomes (not both). Through narrative discussion of the methods and outcomes used in these studies, teamed with concepts from previous literature reviews and evaluation frameworks highlighted, we establish an explicit valuation approach. We propose impact evaluations of OH-interventions (I) establish stakeholder objectives, (II) establish quantifiable outcomes that feed into those objectives, (III) establish agents and compartments that affect these outcomes and (IV) select appropriate methods accordingly (detailed in this paper and within the individual study summaries found in Appendix D).

Through applying our suggested approach to the case study of AMR, we propose evaluations of AMR-interventions take a multi-level compartmental modelling approach. However, this is based on assumed objective functions from the authors, and was not defined through stakeholder participation, which is recommended for future work. In future evaluations, it may be determined from initial stakeholder involvement that the proposed outcome measures of this work are not wholly applicable. In this instance, the literature review's narrative results can still act as a resource for alternative methods used in other evaluations of cross-sectoral interventions' impact.

In terms of generalisability, our approach can be applied across different OH intervention evaluations. Furthermore, as our definition of the system and theory of change used in constructing the proposed conceptual model for evaluation is generic and simplified, it can be adapted and built on to suit local needs for AMR-intervention evaluation. For example, additional compartments related to nutrition-associated health states could be added if it is thought the AMR-intervention could affect food availability or farmer income.

In terms of comparing the findings from the review to similar published evidence, we found around a third of individual studies had both monetary and non-monetary outcomes, which is line with another review focused on outcomes of OH interventions (which also found around one third of studies to have both outcome types) [14] . Though a limitation of both is the focus on quantitative outcome assessment, as opposed to including also qualitative ones. However, this distinction is needed as methods for establishing the former are quite different from the latter.

Our scoping review had limitations. Our chosen method allowed for targeted searches relating to relevant areas (such as climate change literature [18]) for a narrative discussion of methods, but subsequently means that that quoted study proportions of OH issues should not be taken as robustly representative of the total research space. Additionally, literature review results were included as outputs themselves, and individual studies from those literature reviews were not then re-data-extracted, as we wanted to focus on summarising information without duplication of effort. However, this limits the generalisability of presented descriptive statistics across the total research space. Finally, the conceptual model for AMR attempts to simplify the modelling procedures, however, a lot of data, time and monetary resources are needed to apply the proposed model in practice.

In conclusion, defining not only the system and theory of change, but stakeholder objective functions and associated outcomes is needed for impact evaluations of OH interventions to be useful in stakeholder decision making. Compartmental modelling (utilising outputs from statistical analyses of data and inputs from stakeholders) combined with MCDA can allow for AMR interventions to be quantitatively evaluated in a way that maximises evaluation utility for decision-makers across the OH system.

## Funding

This research was funded by, and is a contribution to, the CGIAR Research Program on Agriculture for Nutrition and Health (A4NH). GMK was supported by a fellowship from the UK 10.13039/501100000265Medical Research Council (MR/P014658/1). The opinions expressed here belong to the authors, and do not necessarily reflect those of A4NH or CGIAR. The funder was not involved in the study design, execution or write up processes.

## Declaration of Competing Interest

The authors declare they have no conflicts of interest.

## Footnotes

1 The remainder of the 73 did not state a time horizon or it was not applicable.
